# Evaluation of sleep position shifts in patients with obstructive sleep apnea syndrome with the use of a mandibular advancement device

**DOI:** 10.3389/fdmed.2025.1524334

**Published:** 2025-03-05

**Authors:** Domenico Ciavarella, Donatella Ferrara, Carlotta Fanelli, Graziano Montaruli, Giuseppe Burlon, Michele Laurenziello, Lucio Lo Russo, Fariba Esperouz, Michele Tepedino, Mauro Lorusso

**Affiliations:** ^1^ Department of Clinical and Experimental Medicine, University of Foggia, Foggia, Italy; ^2^ Department of Biotechnological and Applied Clinical Sciences, University of L’Aquila, L’Aquila, Italy

**Keywords:** obstructive sleep apnea (OSA), apnea-hypopnea index (AHI), oxygen desaturation index (ODI), sleep position shifts, mandibular advancement device (MAD)

## Abstract

**Background:**

The aim of this study was to evaluate position shifts during sleep of patients with obstructive sleep apnea (OSA) syndrome both with and without the use of a mandibular advancement device (MAD).

**Methods:**

In total, 73 adult Caucasian patients diagnosed with obstructive sleep apnea syndrome confirmed by polysomnography were retrospectively enrolled. Inclusion criteria were as follows: age >20 years, body mass index <34 kg/m^2^, polysomnographic diagnosis of OSA, non-smoker, absence of comorbidities at diagnosis, and treatment with a MAD. Two polysomnographic monitoring were performed: one at the time of diagnosis (T0) and another after 3 months of treatment (T1). The parameters evaluated were the apnea-hypopnea index, oxygen desaturation index, the total number of position shifts, and position shift index (number of shifts per hour). Since the variables failed the normality test, the Wilcoxon test was performed to analyze the correlation between the mean of polysomnographic parameters at T0 and T1. The difference between the T1 and T0 values for each variable was evaluated using Spearman's rho correlation test. Statistical significance was set at *p* < 0.05.

**Results and conclusions:**

All the parameters, including respiratory and positional measures, were significantly reduced after the use of a MAD compared to the beginning. Spearman’s correlation test revealed a relationship between the total number of sleep position shifts and the sleep position shift index with the oxygen desaturation index. However, no significant correlation was observed between the apnea-hypopnea index and the positional values.

## 1 Introduction

Obstructive sleep apnea syndrome (OSAS) is a sleep-related breathing disorder characterized by repeated episodes of partial or total obstruction of the upper airway during sleep, thus leading to phenomena defined as hypopnea and apnea. An apnea episode, by definition, is the cessation of breathing for at least 10 s. Hypopnea is defined as a reduction in airflow of at least 50%, associated with a reduction in oxygen saturation of >4%. The apnea-hypopnea index (AHI), i.e., the total number of hypopneas and apneas per 1 h of sleep, is used to indicate the severity of obstructive sleep apnea (OSA). There are different categories of sleep apnea depending on the OSA index: normal sleep has an AHI of fewer than 5 events, mild sleep apnea has an AHI of 5–15 events, moderate sleep apnea has an AHI of 15–30 events, and severe apnea has an AHI of more than 30 events per hour (1–3).

### 1.1 Epidemiology

The prevalence of OSA is approximately 22% in men and 17% in women with a gender distribution of 2:1 (4). This distribution is perhaps related to different hormonal effects that induce an increase in upper airway muscle collapsibility, body fat distribution, and different anatomy. Hormonal effects have an important role in OSA pathogenesis, particularly in post-menopausal women compared to pre-menopausal women. Unfortunately, the role of hormones in OSA pathogenesis is still unclear (5).

### 1.2 Comorbidities

Obstructive sleep apnea syndrome is becoming an increasingly studied condition because of its many comorbidities and consequences, although there is a high prevalence of undiagnosed and untreated patients (6). Because of sleep deprivation and daytime sleepiness, patients with OSA have a higher risk of car accidents (7). Cardiovascular disease is correlated with OSA, with sympathetic activation, oxidative stress, and systemic inflammation defined as the main causes of this association. OSA is an independent risk factor for hypertension, coronary artery disease, heart failure, cardiovascular and cerebrovascular diseases (CVDs), and atrial fibrillation (8, 9). OSA is related to many metabolic complications such as type 2 diabetes mellitus (T2DM) (10). The prevalence of T2DM in patients with OSA is higher than in the general population (11). Current research suggests that arousals and sleep fragmentation may have effects on systemic inflammation, sympathetic surges, glucose intolerance, β-cell dysfunction, and insulin resistance (12). Narkiewicz et al. (13) suggested an alternative way to explain the relationship between T2DM and OSA. Oxyhemoglobin desaturation and hypercarbia may alter epinephrine, norepinephrine, and cortisol secretion, which leads to increased gluconeogenesis and decreased glucose uptake.

### 1.3 Symptoms and diagnosis

Sleep fragmentation in patients with sleep apnea can also lead to neurocognitive and behavioral consequences (14, 15). Although there are questionnaires and several risk factors (age >40 years, male sex, obesity, smoking) and symptoms (snoring, nocturia, nocturnal gasping, daytime sleepiness) to identify patients with obstructive sleep apnea, the diagnostic standard to diagnose the condition is nocturnal polysomnography (16, 17). OSA is evaluated by many questionnaires that focus on daytime sleepiness and health-related quality of life (HRQoL) (18, 19). The Epworth Sleepiness Scale (ESS) and the STOP-Bang and Berlin questionnaires are the main questionnaires used to evaluate daytime sleepiness (20, 21). Other questionnaires investigate the HRQoL of patients with OSA [i.e., short form 36 health survey questionnaire (SF36), short form 12 health survey questionnaire (SF12), sleep apnea quality of life index (SAQLI), functional outcomes of sleep questionnaire (FOSQ), and OSA wellness scale (OWS)] (22). A complete overnight sleep test, i.e., polysomnography (PSG), is conducted to evaluate OSA severity. PSG evaluates at least seven different physiological signals. PSG is the “gold standard” in objective-based sleep studies. There are four levels of sleep studies:

 1. Type 1: full attended polysomnography (≥7 channels) in a laboratory setting;
 2. Type 2: full unattended polysomnography (≥7 channels);
 3. Type 3: limited channel devices (usually using 4–7 channels);
 4. Type 4: 1 or 2 channels, usually with oximetry as one of the parameters (23).

The home sleep apnea test (HSAT) is the most frequently used test to reduce the patient's discomfort and provide the most effective evaluation of OSA. The HSAT is a type 3 level of evaluation and gives information to screen the patients in aspects such as oximetry, respiratory monitoring [(a) effort, (b) airflow, (c) snoring, (d) end-tidal CO_2_, and (e) esophageal pressure], cardiac monitoring [(a) heart rate or heart rate variability and (b) arterial tonometry], measures of sleep-wake activity [(a) electroencephalography and (b) actigraphy], body position, and other (24).

### 1.4 Treatment

Continuous positive airway pressure (C-PAP) is the first-line treatment for patients with obstructive sleep apnea. It is a non-invasive treatment method used to maintain airway patency by delivering constant airway pressure. Other alternative methods, such as oral appliances, are also used in patients with apnea, especially for those who do not tolerate the C-PAP mask. Surgery is only used in cases with anatomic obstructions that need to be corrected (25). The oral appliances used are tongue retainer devices (TRDs) and mandibular advancement devices (MADs). A TRD is made of a flexible material with a bulb-like receptacle in the anterior portion. It maintains the tongue in a forward position during sleep, reducing stress on the upper airway and against the posterior pharyngeal wall (26). A MAD has been recommended by the American Academy of Sleep Medicine as a treatment for mild to moderate OSA (27). Some studies suggest that it also has an excellent effect on severe apnea. A MAD is a device with important advantages: low cost, simple production, and portability (28). The effect of a MAD is to increase the upper airway space through a forward and vertical movement of the jaw along with a repositioning of the hyoid bone and tongue (29). A MAD is built as two occlusal splints, fully covering the teeth, allowing for an increase in mandibular sagittal movement and free vertical/transversal movement of the jaw (30). The role of the dentist is becoming increasingly important for both the diagnosis and treatment of snoring and obstructive sleep apnea (31).

### 1.5 Research aim

It is well-known that obstructive sleep apnea causes restless sleep, tossing and turning, circadian misalignment, and daytime sleepiness. Therefore, sleep deficiency and poor sleep quality are closely linked to OSA (32, 33). OSA is associated with several other sleep disorders (e.g., insomnia and restless legs) and sleep-related problems [e.g., excessive daytime sleepiness (EDS)] (34).

In the International Classification of Sleep Disorders second edition (ICSD-2), the IV group describes the most common symptoms of dyssomnias (insomnia and EDS) and parasomnias (abnormal physiological events) such as sleep-related movement disorders (SRMDs), restless legs syndrome, periodic limb movement disorder, sleep-related leg cramps, and sleep-related bruxism (35). Sleep-related movement disorders involve characteristic body movements that alter sleep. When these disorders coexist, there is an increase in cumulative morbidity, and they are likely to negatively affect each other (36).

Sleep position has already been associated with sleep apneas, defined in most studies in the literature as an increase in AHI in the supine position, later coming to define positional apneas and subsequent positional therapy (37). Restless sleep, however, and constant position changes have been less investigated in the literature, except for the association with other types of pathologies.

A modification of nervous activation may be correlated to the night shifts in sleep time, as documented during the periodic limb movements during sleep (PLMS) and other SRMDs. PLMS are repetitive, stereotypical limb movements that can lead to arousals and sleep fragmentation. PLMS are also more prevalent among patients with OSA than in the general population (38). PLMS are strongly associated with increased sympathetic activity. The correlation between the PLMS and sympathetic activation is still debated (39). The sympathetic activation may be due to PLMS-triggered arousal. It is well-known that sympathetic activation is correlated to blood pressure, vascular inflammation, hypercoagulation, and dyslipidemia (40, 41).

Khan hypothesized that an increase in position shifts during sleep (PSDS) may be correlated with a modification of the nervous system activation, as PLMS and SRMD treatment with a reduction of movement is correlated with a reduction of sleep arousals and cardiorespiratory efforts (42). The effects of oral treatment on OSA and SRMDs are still a controversial issue. Bariani et al. (43) showed that rapid maxillary expansion (RME) treatment had effects in children with OSA and PLMS. Other research studies have tried to evaluate the effects of a MAD on PLMS. The effect of a MAD on PLMS was to reduce the body movement events, similar to C-PAP treatment (44). In the present paper, the authors evaluated the effect of MAD treatment on patients with mild to severe OSA, focusing on cardiorespiratory effects [i.e., AHI and oxygen desaturation index (ODI)] and position shifts during sleep. The frequency of position shifts during sleep may be correlated to stress during sleep caused by the number of apnea events and oxygen desaturation. Using the HSAT, the present study aimed to investigate the correlation between position shifts during sleep caused by OSA and their modification due to treatment with a MAD.

## 2 Materials and methods

### 2.1 Study population

This study was reported following the Strengthening the Reporting of Observational Studies in Epidemiology (STROBE) guidelines for observational studies (45). This retrospective study involved 73 adult Caucasian patients (43 men and 30 women; mean age 46 ± 4 years) selected from patients with OSA treated at the Department of Orthodontics, University of Foggia, Italy. All patients consented to be included in the present research. Patients were selected using the following inclusion criteria: age >20 years, body mass index <34 kg/m^2^, diagnosis of OSA by nocturnal polysomnography, absence of comorbidities at the time of diagnosis, non-smoker, treatment with a mandibular advancement device, and treated from February 2021 to March 2023. All the procedures described in the present research protocol adhered to the Declaration of Helsinki (1975) (and the subsequent revisions) and were approved by the Ethics Committee of the University of Foggia (Approval no. 43/CE/2019) and all patients gave their informed consent to participate. A power analysis (G*Power 3.1.9.2, Franz Faul, Universitat Kiel, Germany) revealed that to detect a large effect size of 0.5 (46) with the Wilcoxon signed-rank test, an α error probability of 0.05, and a power (1 − β error prob) of 0.95, 47 participants would be needed.

### 2.2 Methods and parameters

All patients underwent drug-induced sleep endoscopy (DISE) to assess their upper airway closure type and position. A split-night polysomnogram test (SN-PSG) was conducted in a sleep laboratory for each patient before treatment (T0) using a type 2 portable device (Embletta X-100 system, Flaga, Reykjavik, Iceland) that recorded electroencephalograms, electrooculograms, electromyograms, pulse oximetry channels, abdominal respiratory effort bands, body position sensors, nasal cannulas, and oral thermistor. Another SN-PSG was conducted after 3 months of treatment with a mandibular advancement device (T1). The parameters extracted from the night records were the following: AHI, ODI, the number of position shifts (NPS), and the position shifts index (PSI) (Table 1).

**Table 1:** Description of the indices recorded during the overnight polysomnography.

Polysomnographic evaluation	Description
AHI	Number of apnea and hypopnea events per hour of sleep
ODI	Number of oxygen desaturations
NPS	Number of sleep body movements
PSI	Number of sleep body movements/hour

ODI was calculated based on oxygen desaturation being lower than 3%, as recommended by the American Academy of Sleep Medicine (47).

The primary outcome of the study was to evaluate changes in polysomnographic indices and positional shifts after treatment with a MAD. The secondary outcome was to analyze the correlation between positional changes and polysomnographic indices.

**Figure 1:**
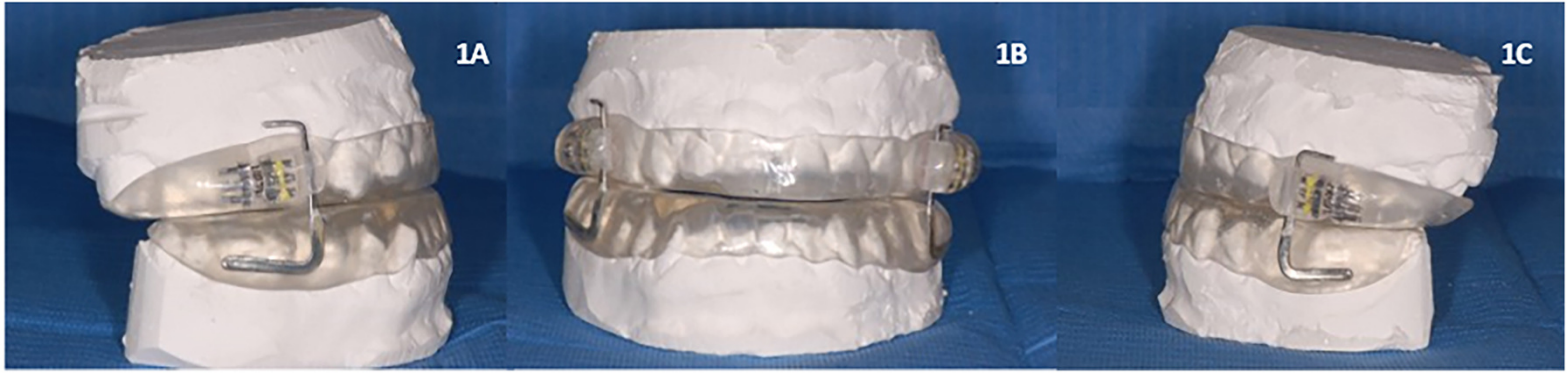
IMYS device (It Makes You Sleep). **(A)** Right lateral view, **(B)** frontal view, and **(C)** left lateral view.

**Figure 2:**
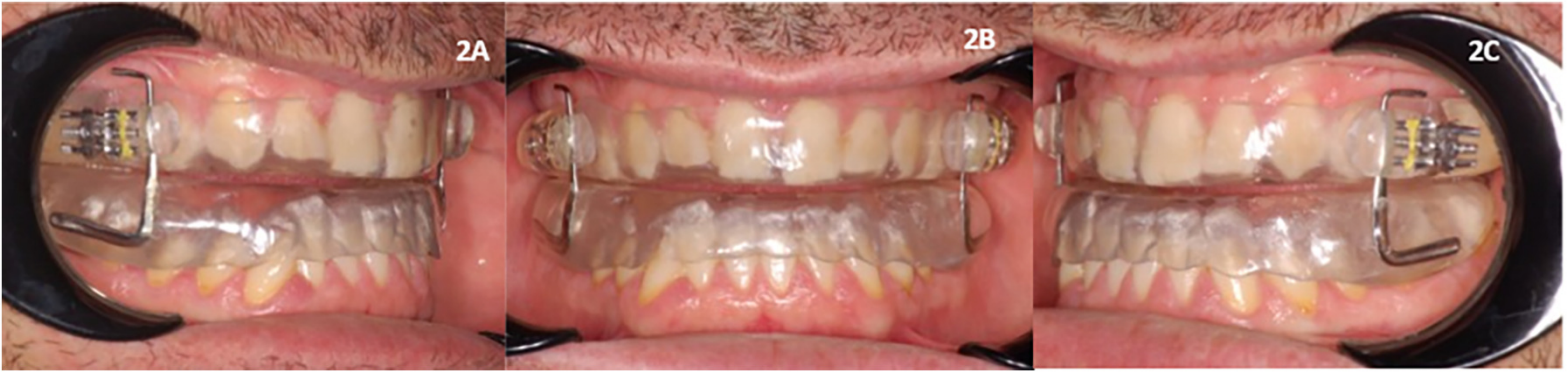
IMYS device (It Makes You Sleep) in a patient's mouth. **(A)** Right lateral view, **(B)** frontal view, and **(C)** left lateral view.

**Figure 3:**
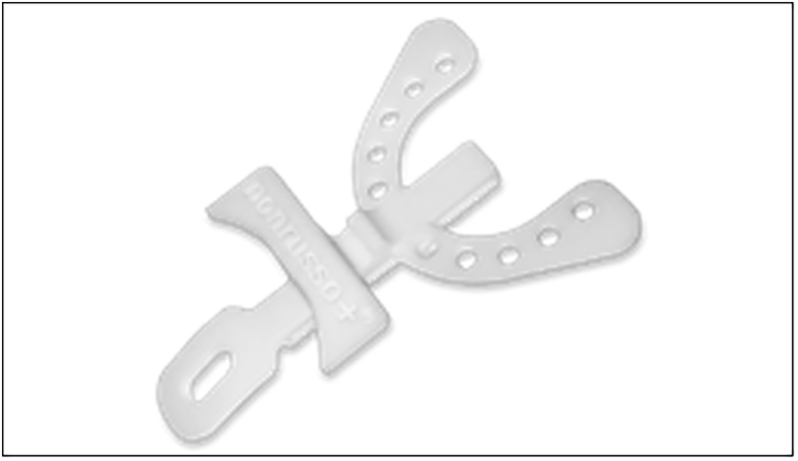
The OCCLUSION® device was used to register the ideal mandibular protrusion and to transfer the desired mandibular position to the dental laboratory for the manufacturing the MAD device.

The device used was a customized and adjustable device called IMYS (It Makes You Sleep), ideated by Professor D. Ciavarella (Figures 1, 2). It consists of two resin splints connected by two vertical stainless-steel bars and two lateral screws, and the inclusion of these components facilitated the adjustment of mandibular advancement. The design of the vertical arms had a top end that fitted into a vertical space (mesial to the screw) set in the resin of the upper splint, and a bottom end incorporated in the resin of the lower splint. These arms allowed a slight vertical mandibular movement, preventing a full mandibular opening. Moreover, the vertical space in which the upper arm was set, enabled slight lateral movements for the mandible. To enhance the tongue's position, a vertical palatal spot was added to the upper splint (29). A functional mandibular evaluation was conducted on each patient. An irreversible hydrocolloid material (Orthoprint Zhermack®) was used to take the impressions of both the upper and lower arches of each patient, employing non-perforated Rim Lock trays. Cast models were generated through impressions using high-strength type IV dental stone (such as Fujirock, Vel-Mix, and Suprastone), and these models were subsequently mounted on an articulator. An initial advancement of 70% of the total was established for each patient using an intraoral gauge (Occlusion®, Nonrusso+®, Dr. Giuseppe Burlon, Belluno, Italy) (Figure 3). The occlusion gauge (OG) features a millimetric grid for assessing sagittal mandibular shifts, along with engravings for both upper and lower incisors. The lower part of the gauge was designed to allow for sagittal movement. Before recording the effective mandibular protrusion, patients were instructed to move their jaw forward and backward. An initial advancement of 70% of the total mandibular movement, both backward and forward, was applied. The occlusion position with 70% activation using Occlufast [Zhermack Spa, Via Bovazecchino, 100–45021 Badia Polesine (RO), Italy] was then recorded. Every 2 weeks, the MAD was adjusted in increments of 0.25 mm to achieve the most functional and comfortable position for the patient.

Patients used the device overnight for at least 8 h.

### 2.3 Statistical analysis

Data distribution analysis was conducted using the Shapiro–Wilk normality test. Descriptive statistics were also performed. Because the variables failed the normality test, a Wilcoxon signed-rank test was used for comparison of the polysomnographic parameters taken at T0 (pre-treatment) and T1 (3 months after treatment). Spearman's rho correlation test was performed to assess the relationship between the polysomnographic variables at T1 and T0. The significance index was set to *p* < 0.05. Data were analyzed using GraphPad Prism software 6.0 (GraphPad Prism Software, San Diego, CA, USA).

## 3 Results

Table 2 shows the polysomnographic data distribution of all variables before and after MAD treatment. The patients had mild OSA overall (mean AHI 25.2 e/h and mean ODI 18.8 e/h). The mean NPS was 479.2 and the mean PSI was 69.56 (Table 2). Following MAD treatment, a reduction of both the AHI (−16.36 e/h, *p* < 0.01) and ODI (−9.556 e/h, *p* < 0.01) was observed (Table 2).

After MAD treatment, a decrease in the number of sleep shifts was observed, with NPS decreasing by −402.5 (*p* < 0.01) and PSI decreasing by −58.35 (*p* < 0.01) as indicated in Table 2.

The authors evaluated the correlation between NPS and PSI (difference between T1 and T0) with the AHI and ODI (T1 and T0). The test demonstrated that the reduction in the ODI was correlated with both NPS (rho 0.447 NPS to ODI, *p* < 0.01) and PSI (rho 0.416, *p* < 0.01). No statistical correlation between positional indicators (i.e., NPS and PSI) and the AHI was observed (NPS to AHI: rho 0.216, p = n.s.; PSI to AHI rho 0.182, p = n.s.) (Table 3).

**Table 2:** Descriptive statistics, normality test, and Wilcoxon signed-rank test for polysomnographic variables (*n* = 73) before treatment with a MAD (T0) and after treatment (T1).

Descriptive statistics	AHI T0 (e/h)	AHI T1 (e/h)	ODI T0 (e/h)	ODI T1 (e/h)	NPS T (events)	NPS T1 (events)	PSI T0 (e/h)	PSI T1 (e/h)
Mean	25.2	9.30	18.8	7.10	479.2	73.88	69.56	12.18
Std. deviation	11.4	10.7	10.0	9.98	111.8	171.8	80.73	17.93
Median	23.6	6.05	18.7	3.50	108.0	13.85	39.50	5.95
Lower 95% CI of the mean	21.0	5.43	15.2	3.51	76.20	11.95	40.45	5.71
Upper 95% CI of the mean	29.3	13.1	22.5	10.7	882.2	135.8	98.67	18.64
Difference	−16.35		−9.556		−402.5		−58.35	
*p*-value (Wilcoxon test)	**		**		**		**	

** *p* < 0.01.

**Table 3:** Spearman's rho correlation test between the patient's sleep movement and the polysomnography indexes (*n* = 73).

Variables	NPS	PSI	AHI	ODI
NPS		0.975°	0.216°	0.447**
PSI	0.975°		0.182°	0.416**
AHI	0.216°	0.182°		0.767°
ODI	0.447**	0.416**	0.767°	

° n.s., **p < 0.01.

## 4 Discussion

Recently, OSA has increasingly attracted interest because of its serious health impact, particularly the association between obstructive sleep apnea and increased cardiovascular risk. OSA has been defined as an independent risk factor for hypertension, stroke, coronary artery disease, heart failure, and arrhythmias (8, 9, 48). Moreover, there is much evidence supporting the association between OSA and metabolic disorders such as diabetes and impaired glucose control (12, 49, 50). In addition, repeated arousals and sleep fragmentation can lead to neurocognitive and mood deterioration including depression and anxiety (51). Daytime sleepiness may affect daily activities by reducing performance and causing occupational accidents, injuries, and car accidents (7, 52). In this context, the treatment of obstructive sleep apnea becomes crucial for the patient's health. Although C-PAP is still the treatment of choice for sleep apnea therapy, the use of mandibular advancement devices is increasingly common. A MAD ensures mandibular protrusion to keep upper airways open during sleep, improving oxygenation; reducing the number of apneas, hypopneas, and arousals; and improving the associated subjective and objective symptomatology (30, 53). Therefore, there is substantial evidence demonstrating the efficacy of these devices. The present study highlighted a new feature of MADs: the ability to reduce the number of shifts during sleep. Sleeping position and body posture are closely linked to the occurrence and severity of sleep apnea (54, 55). The body's nocturnal movements are linked to brain arousal. Nevertheless, it remains unclear whether the number of position shifts during sleep can influence arousals and daytime quality of life (22). Zhang et al. (56) monitored 13 subjects, without sleep disorders, using a sleeping-position monitoring device to determine the impact of sleeping positions and turning shifts on sleep quality. They found that patients with a higher turning frequency had poor sleep quality. De Koninck et al. (57) agreed with one of the initial studies on sleep and body position conducted by the Health Physics Society (58), finding that poor sleepers had more frequent position changes during sleep compared to good sleepers, who spent more time in the same position. The present study correlates the high number of position shifts during sleep in patients suffering from apnea with OSA severity. Although it is common to change positions several times during sleep, the number of shifts appears to vary among individuals and differs among specific groups. According to Skarpsno et al. (59), the number of shifts during sleep is higher in young people than in older people. In addition, women experience fewer nocturnal movements compared to men, and a high BMI is associated with fewer shifts in sleep position. Skarpsno et al. reported an average number of 1.6 position shifts per hour, with 1.77 in men and 1.36 in women. The mechanisms underlying the frequency of positional changes during sleep are not fully understood. Tossing and turning generally occur during “arousals”. It 's possible that cortical signals swiftly transition from the sleep state to waking actions, causing the person to change positions and resume sleep without remembering the position shift. Sleep fragmentation is a common condition in sleep breathing disorders such as OSA (17). On the one hand, arousal is an important lifesaving mechanism to overcome narrowing and stabilize breathing, but, on the other hand, it is associated with increased activity of the sympathetic nervous system (60). PLMS is a movement disorder that causes repetitive and involuntary arm movements during sleep.

This disorder is commonly linked to other conditions such as Parkinson's disease and narcolepsy. In such cases, it is referred to as secondary PLMDS. However, at times, it is not associated with other diseases and has no known cause, leading to its classification as primary PLMDS. The literature indicates that patients with OSAS often suffer from PLMS. The mechanisms have not yet been fully discovered; an over-activation of the sympathetic system is likely an underlying factor. However, OSAS patients with PLMS tend to be older, have shorter rapid eye movement (REM) duration, and have a higher AHI (60). All these characteristics of patients with OSA may be predisposing factors for restless sleep and an increased number of sleep positions. Consequently, sleep quality in OSA patients also seems to be associated with the number of position changes during sleep.

The number of sleep movements may impact patients' sleep quality, directly affecting their quality of life and cardiovascular function. The authors evidenced how OSA led to significant changes in patients' nightly body positions, which were correlated with their ODI. This aspect needs further clarification due to its potential relevance to factors such as daytime sleepiness, nocturnal agitation, and heart rate modifications.

### 4.1 Limitations of the study

The limitation of this study is due to the retrospective nature of patient recruitment, although care was taken to avoid any selection bias thanks to the use of a rigid chronological criterion. Due to the retrospective nature of this study, it is difficult to understand which other unanalyzed variables might have influenced the relationship between the polysomnographic variables and the MAD effects. Since only one type of MAD was used, the results obtained could differ if other devices were used. Future studies should implement a longitudinal case–control design and a long-term follow-up.

## 5 Conclusions

In the present paper, the authors showed how MAD treatment may modify the severity of OSA by reducing polysomnographic parameters (i.e., AHI and ODI). In addition, a mandibular advancement device has the capability to decrease the number of positional shifts during sleep, resulting in an improved quality of sleep.

In this paper, the reduction of respiratory effort events was correlated with a decrease in patient night shifts. The conclusions regarding patients with OSA treated with a MAD are as follows:

• a reduction in the AHI and ODI;
• a reduction of NPS and the PSI;
• a statistical correlation between the reduction of the ODI and NPS;
• a statistical correlation between the reduction of the ODI and PSI.

Position shifts are yet another consequence of the poor sleep quality experienced by patients with sleep apnea and a MAD may be the key to achieving restorative sleep.

## Data Availability

The raw data supporting the conclusions of this article will be made available by the authors, without undue reservation.
