# Corrigendum: Evaluation of sleep position shifts in patients with obstructive sleep apnea syndrome with the use of a mandibular advancement device

**DOI:** 10.3389/fdmed.2025.1593050

**Published:** 2025-05-14

**Authors:** Domenico Ciavarella, Donatella Ferrara, Carlotta Fanelli, Graziano Montaruli, Giuseppe Burlon, Michele Laurenziello, Lucio Lo Russo, Fariba Esperouz, Michele Tepedino, Mauro Lorusso

**Affiliations:** ^1^ Department of Clinical and Experimental Medicine, University of Foggia, Foggia, Italy; ^2^ Department of Biotechnological and Applied Clinical Sciences, University of L’Aquila, L’Aquila, Italy

**Keywords:** obstructive sleep apnea (OSA), apnea-hypopnea index (AHI), oxygen desaturation index (ODI), sleep position shifts, mandibular advancement device (MAD)

A Corrigendum on Evaluation of sleep position shifts in patients with obstructive sleep apnea syndrome with the use of a mandibular advancement device By Ciavarella D, Ferrara D, Fanelli C, Montaruli G, Burlon G, Laurenziello M, Lo Russo L, Esperouz F, Tepedino M and Lorusso M (2025). Front. Dent. Med. 6:1524334. doi: 10.3389/fdmed.2025.1524334

In the published article, there was an error in Table 3 as published. Incorrect negative values were entered when copying the results into the table. The corrected Table 3 and its caption appear below.

**Table 3:** Spearman's rho correlation test between the patient's sleep movement and the polysomnography indexes (*n* = 73).

Variables	NPS	PSI	AHI	ODI
NPS		0.975°	0.216°	0.447**
PSI	0.975°		0.182°	0.416**
AHI	0.216°	0.182°		0.767°
ODI	0.447**	0.416**	0.767°	

° n.s., **p < 0.01.

In the published article, there was an error. The incorrect values in Table 3 were reported to **Results**, *paragraph 3*.

The corrected sentence appears below:

“Table 2 shows the polysomnographic data distribution of all variables before and after MAD treatment. The patients had mild OSA overall (mean AHI 25.2 e/h and mean ODI 18.8 e/h). The mean NPS was 479.2 and the mean PSI was 69.56 (Table 2). Following MAD treatment, a reduction of both the AHI (−16.36 e/h, *p* < 0.01) and ODI (−9.556 e/h, *p* < 0.01) was observed (Table 2). After MAD treatment, a decrease in the number of sleep shifts was observed, with NPS decreasing by −402.5 (*p* < 0.01) and PSI decreasing by −58.35 (*p* < 0.01) as indicated in Table 2. The authors evaluated the correlation between NPS and PSI (difference between T1 and T0) with the AHI and ODI (T1 and T0). The test demonstrated that the reduction in the ODI was correlated with both NPS (rho 0.447 NPS to ODI, *p* < 0.01) and PSI (rho 0.416, *p* < 0.01). No statistical correlation between positional indicators (i.e., NPS and PSI) and the AHI was observed (NPS to AHI: rho 0.216, *p* = n.s.; PSI to AHI rho 0.182, *p* = n.s.).”

The authors apologize for these errors and state that this does not change the scientific conclusions of the article in any way. The original article has been updated.

